# Editorial: Neuromodulatory Control of Spinal Function in Health and Disease

**DOI:** 10.3389/fncir.2019.00084

**Published:** 2020-01-22

**Authors:** Brian R. Noga, Shawn Hochman, Hans Hultborn

**Affiliations:** ^1^Miller School of Medicine, University of Miami, Miami, FL, United States; ^2^Department of Physiology, Emory University School of Medicine, Atlanta, GA, United States; ^3^Department of Neuroscience, University of Copenhagen, Copenhagen, Denmark

**Keywords:** spinal cord, neuromodulation, monoamines, descending and segmental pathways, sensorimotor systems, locomotion, spinal cord injury, pain

The classical ionotropic transmitters glutamate/ACh (acetylcholine) and glycine/GABA (gamma-amino butyric acid) are, respectively, responsible for the primary excitatory and inhibitory synaptic actions within spinal cord anatomical circuits, be they simple reflexes as the monosynaptic stretch reflex (and its reciprocal inhibition of antagonists), or more distributed and integrated networks along autonomic, sensory, and motor systems. The selection and complex spatiotemporal recruitment of intrinsic spinal circuits (e.g., locomotion) are profoundly sculpted by neuromodulation acting both at pre- and post-synaptic levels.

Neuromodulation denotes to the ability of neurons to alter their electrical and synaptic properties in response to intracellular biochemical changes. Neuromodulation commonly occurs via activation of metabotropic (G protein-coupled) receptors that alter signal transduction pathways. Neuromodulators function to modulate rather than mediate activity and represent a broad class of neuroactive substances. By altering the cellular/synaptic properties of individual neurons embedded in networks, neuromodulators profoundly alter the operation of neural circuits and behavior. They provide flexibility in circuit selection and strength to allow the nervous system to adapt neural output according to the functional requirements and/or demands of the individual to achieve the desired behavioral state. At times, it appears that the neuromodulators have a more primary function in activating and controlling complex networks than the term “modulator” would tend to imply.

Neuromodulatory transmitter systems undoubtedly affect all spinal cord functional systems including locomotion, respiration (via phrenic and other respiratory motoneurons), posture, balance, fine movements, autonomic functions (including control of bowel, bladder, blood pressure, and heart rate), reflexes, and sensory information processing (nociception, etc.). Since many neuromodulatory transmitter systems (e.g., such as those releasing monoamines) originate from neurons outside of the spinal cord, injuries to the spinal cord will necessarily damage their descending projections in addition to other non-neuromodulatory pathways controlling the anatomical network. Such injuries will therefore affect not only the initiation and control of spinal networks, but also the ability to adjust their output according to ongoing functional demands.

The present Research Topic includes review and original research articles that seek to shed light on the neural processes underlying the neuromodulatory control of spinal cord function in health and disease. This Research Topic consists of 24 articles on various aspects of Spinal Cord research contributed by 105 authors. The assembled contributions are summarized below in three thematic categories: (i) locomotion, (ii) descending and segmental pathways, and (iii) disease/injury.

## Locomotion

The mesencephalic locomotor region (MLR) activates spinal locomotor generating neurons via pathways originating in the medial reticular formation and descending through the ventral funiculus. There is evidence that monoaminergic pathways are also activated both during MLR-evoked fictive locomotion and during spontaneous or voluntary locomotion. In the present original research, Noga et al. measured in real time, by fast-cyclic *in vivo* voltammetry, the release of the monoamines serotonin (5-HT) and norepinephrine (NE) in the decerebrate cat's lumbar spinal cord during MLR-evoked fictive locomotion. The time course, the spinal locations and the concentration of the release of these two monoamines were mapped. Monoamine release was observed in dorsal horn, intermediate zone/ventral horn. The results demonstrate that spinal monoamine release is modulated on a timescale of seconds, and that the concentrations are high enough to strongly activate various receptors subtypes and further suggest that monoamine action is, in part, mediated by extrasynaptic neurotransmission in the spinal cord.

The review by Sharples et al. focuses on the role of dopamine in the spinal control of locomotion in vertebrates. It summarizes the biochemistry, pharmacology, anatomy, and function of dopamine (and to a lesser extent other monoamines) in the locomotor networks of various vertebrates including fish (lamprey and zebrafish), amphibians (larval xenopus), and mammalians (rodents). A brief discussion of the effects of dopamine on rhythmicity in invertebrates is also provided. Parallels are drawn with both noradrenergic and serotonergic systems. New experimental approaches (optogenetics, pharmacogenetics) together with more traditional approaches (intrathecal administration/cell transplantation) to the study of dopaminergic function and/or the treatment of gait disorders following SCI are discussed.

Sensorimotor transformations are essential for control of goal-directed behavior and interaction with the outside world. The review by Daghfous et al. examines how the vertebrate CNS integrates sensory signals to generate locomotor behavior by examining the pathways and mechanisms involved in the transformation of cutaneous and olfactory inputs into motor output in the lamprey. The review also explores how serotonin modulates the system through actions on both sensory inputs and motor output. This timely review of mechanisms for sensory control of locomotion is also very thorough and helpful to the uninitiated in this area of lamprey pharmacology.

Wienecke et al. studied the interaction between blood pressure, respiration, and locomotor activities in decerebrate cats. They observed periodic variation (Mayer waves) in blood pressure, which was synchronized with respiratory and locomotor drive potentials recorded in hindlimb motoneurons. The report demonstrates the intricate interrelations between respiratory networks and hindlimb locomotor networks. They conclude that the respiratory drive in hindlimb motoneurons is transmitted via elements of the locomotor central pattern generator. The rapid modulation related to Mayer waves suggests the existence of a more direct and specific descending modulatory control than has previously been demonstrated. The possibility that monoaminergic drive to the spinal cord may mediate the occurrence of Mayer waves is discussed.

In addition to the classical monoamine neuromodulatory transmitters, another group of endogenous monoamines, the trace amines (TAs), may play a role in neuromodulatory actions within the nervous system. They share structural, metabolic, physiologic, and pharmacologic similarities to the classical monoamines and are synthesized from the same precursor amino acids. The paper by Gozal et al. provides evidence for the presence of trace amine synthetic enzymes, trace amine-associated receptors (TAARs), and TAs in the spinal cord. Furthermore, the authors demonstrate effects of TAs on spinal neuronal networks, effects that have a pharmacological profile distinct from that of more classical monoamine neurotransmitters. This data indicates that TAs may function as an intrinsic spinal monoaminergic modulatory system capable of promoting recruitment of locomotor circuits independent of the descending monoamines. These actions support their known sympathomimetic function. This study will likely stimulate further research in this area.

How pattern generators are modulated in the absence of descending control from the brain is highly important for understanding the neural control of movement as well as for developing therapeutic approaches to improve mobility of SCI patients. The review by Cherniak et al. summarizes recent studies of sacral relay neurons with lumbar projections and evaluates their role in linking the sacral and thoracolumbar networks during different motor behaviors. They show that: (1) the activation of the locomotor central pattern generators through sacral sensory input is mediated by a heterogeneous group of dorsal, intermediate and ventral sacral-neurons with ventral and lateral ascending funicular projections, and (2) the rhythmic excitation of lumbar flexor motoneurons, produced by exposing the sacral segments to alpha-1 adrenoceptor agonists, is mediated exclusively by ventral clusters of sacral-neurons with lumbar projections through the ventral funiculus. The mechanisms and physiological implications of their findings are discussed. Given the recent increase in interest in combinatorial approaches to increasing function after spinal injury, this review of neural mechanisms contributing to the sacro-caudally activated lumbar motor rhythm is timely and should be of general interest to spinal cord rehabilitation, plasticity, and regeneration researchers.

The monoamines serotonin (5-HT) noradrenaline (NA) and dopamine (DA) are known to reconfigure spinal circuits and facilitate expression of motor rhythms including locomotion. Beliez et al. explore the common and differential actions of these monoamines in coordinating rhythmic locomotor-related output along the thoracolumbar-sacral axis in the *in vitro* isolated neonatal rat spinal cord. The monoamines generated similar ventral root motor rhythms in terms of period/duration as well as left/right and flexor/extensor phase relations but differed in motor burst amplitude and other temporal characteristics including intersegmental phase relationships. Observed differences likely relate differences in descending behavioral drive linked to separate recruitment of these neuromodulators.

## Descending and Segmental Pathways

C-boutons are important cholinergic modulatory loci for state-dependent alterations in motoneuron firing rate. Deardorff et al. present an elegant and systematic review of the state of knowledge of one of the major inputs to spinal motoneurons, the C terminals. The authors take the reader on a historical journey from the initial identification of the C-boutons and then introduce the unique molecular organization of the signaling ensemble surrounding the C-bouton synapse and its effect on the firing frequency of the spinal motoneuron via its effect on the post-spike after hyperpolarization. They then describe the C-bouton itself and its cell of origin, and its cholinergic identity and circuitry. Finally, they propose a possible mechanism by which the activity of C-bouton may play a role in enhancing firing rate during periods of increased excitatory drive, whilst also acknowledging alternative explanations.

Bulbospinal systems may influence spinal neurons by classical synaptic and modulatory mechanisms and are involved in motor, sensory, and autonomic functions. Huma et al. report on the brainstem locations of cells of origin of bulbospinal pathways of the rat passing through the medial longitudinal fasciculus (MLF) and the caudal ventrolateral medulla (CVLM). Neurons were identified using anatomical tracing methods, their transmitter phenotypes identified, and their locations mapped onto brainstem diagrams. Cells that form pathways from the brainstem to the lumbar spinal cord passing through the MLF and CVLM for the most part, have overlapping spatial distributions. Although both populations contain crossing and uncrossing axons and similar proportions of excitatory and inhibitory axons, MLF and CVLM reticulospinal neurons have different spinal cord projections. Those in the MLF project more ventrally and are more likely to have direct motor functions than those in the CVLM. In contrast, CVLM projections are predominantly ipsilateral and concentrated within deep dorsal horn and intermediate gray but do not extend into motor nuclei or lamina VIII. CVLM pathway may function to coordinate activity of premotor networks.

Johnson and Heckman provide a comprehensive review of the neuromodulatory control of the electrical properties of spinal motoneurons. Gain control of motoneuron output is important for generating the enormous range of forces required for the wide dynamic range of the normal movement repertoire. For diffuse neuromodulatory systems such as the monoaminergic projection to motoneurons, independent control of the gains of different motor pools is not feasible. In fact, the system is so diffuse that gain for all the motor pools in a limb likely increases in concert. Additionally, if there is a system that increases gain, probably a system to reduce gain is also needed. In this review, they summarize recent studies that show local inhibitory circuits within the spinal cord, especially reciprocal and recurrent inhibition, have the potential to solve both of these problems as well as constitute another source of gain modulation.

## Disease/Injury

In the manuscript by Becker and Parker, the lamprey model is used to compare cellular and synaptic properties of neurons above and below the lesion site in spinal cord injured (SCI) animals and in normal spinal cord of uninjured animals. They also examined the effects of lesioning on the modulatory effects of 5-HT. Based on their results, the authors suggest lesion specific changes occur in cellular and synaptic properties and in serotonin modulation. Therefore, pharmacological approaches to facilitate functional recovery should not be based on the effects reported in uninjured spinal cords. Although, the cellular and synaptic properties of motor neurons and spinal interneurons caudal to lesion site has been demonstrated previously by the same group, the strong differences between larval and adult stages justified the analysis in young adult lampreys. This is the first investigation on the effects of a spinal lesion on the modulatory effects of serotonin, and thus the study has the potential to make a strong contribution to the related literature.

The original paper by Kou et al. describes the changes in the neural circuits in streptozotocin (STZ)-induced diabetic rats. Diabetic polyneuropathy (DPN) is one of the most common complications of diabetes mellitus but is not a single entity and encompasses several neuropathic syndromes, including sensory and motor defects. In this paper, the authors discuss both progressive mechanical allodynia and impaired locomotor activity. The alterations in myelinated nerve fibers, unmyelinated non-peptidergic nerve fibers, and peptidergic nerve fibers might be involved in the early stages of the development in DPN. The underlying mechanism of DPN might be addressed by the dysfunction of those subpopulations of afferents from the peripheral nervous system to the CNS. Both the allodynia and locomotor defects could be prevented and reversed by intrathecal insulin injection.

The review by Fields and Mitchell addresses the well-known ability to exhibit plasticity of the neural system controlling breathing. The focus of the review is the less appreciated ability to exhibit metaplasticity, i.e., a change in the capacity to express plasticity (“plastic plasticity”). Key examples of metaplasticity in respiratory motor control, and our current understanding of mechanisms giving rise to spinal plasticity and metaplasticity in phrenic motor output is discussed. The metaplasticity is especially seen after pre-conditioning with intermittent hypoxia. This metaplasticity is not confined to the respiratory network but is also seen in motor networks involved in limb movements.

The broad review by Ghosh and Pearse covers the promotion or facilitation of serotonergic signaling (including specific receptor systems) to enhance motoneuron excitability, stimulate CPG activity, and restore locomotor function following spinal cord lesions. These strategies have included pharmacological modulation of serotonergic receptors, through the administration of specific 5-HT receptor agonists, or by elevating the 5-HT precursor 5-hydroxytryptophan, which produces a global activation of all classes of 5-HT receptors. Another approach has been to employ cell therapeutics to replace the loss of descending serotonergic input to the CPG, either through transplanted fetal brainstem 5-HT neurons at the site of injury that can supply 5-HT to below the level of the lesion or by other cell types to provide a substrate at the injury site for encouraging serotonergic axon regrowth across the lesion to the caudal spinal cord for restoring locomotion. This approach is one direction at the forefront of research for generating putative interventional approaches for the treatment of SCI.

Serotonergic systems are important for activation and modulation of locomotor circuits. However, locomotor circuits function differently following SCI that damages descending serotonergic (and other) pathways. The study by Strain et al. examines how the spinal cord adapts to sensory perturbations after injury when the serotonergic system is activated. They observed differences in the intralimb and interlimb coordination in SCI animals during serotonergic agonist-induced locomotor movements following sensory perturbation (range-of-motion restriction) in comparison to that seen in intact animals. Differences were observed for hindlimb and forelimb locomotor movements controlled by the distal and proximal (to spinal transection) areas of the spinal cord, respectively. The number of hindlimb steps observed following quipazine treatment was also significantly greater than intact controls, suggesting that the effects of quipazine treatment are related to “supersensitivity” of spinal segments distal to the site of injury. The results have implications for design of rehabilitation strategies to treat paralysis following SCI.

The central molecular changes that might influence neurotrophic signaling pathways and modulate locomotor recovery and pain following SCI are investigated in the study by Strickland et al. Based on their previous work, they assess the expression of select miRNA species that might influence neurotrophic signaling pathways and functional recover following noxious peripheral electrical stimulation. The data show that uncontrollable nociception which activates sensorimotor circuits distal to the injury site, influences SCI-miRNAs and target mRNAs within the lesion site. SCI-sensitive miRNAs may well mediate adverse consequences of uncontrolled sensorimotor activation on functional recovery. However, their sensitivity to distal sensory input also implicates these miRNAs as candidate targets for the management of SCI and neuropathic pain.

The long-term effectiveness of opiates for the treatment of pain is limited by the development of tolerance. This is thought to be the result of dysfunction of the μ-opioid receptor (MOR) and dopamine (D) receptor mediated second messenger pathways in the brain. Brewer et al. examine the role of the spinal cord in the development of tolerance since it plays a prominent role in the processing of nociceptive information and has both dopamine and MOR receptors in the dorsal horn. They reconfirm that D3 receptors are necessary for morphine analgesia *in vivo* and show for the first time that acute block of D3 receptors in the lumbar spinal cord prevents modulation of spinal reflex amplitude by morphine *in vitro*. Their data suggest that the D3 receptor modulates the MOR system in the spinal cord, and that a dysfunction of the D3 receptor can induce a morphine-resistant state. They propose that the D3KO mouse may serve as a model to study the onset of morphine resistance at the spinal cord level, the primary processing site of the nociceptive pathway.

Involuntary movements and spasms may be the result of hyperactive motor networks. Regulation of such networks may be accomplished by intrinsic modulatory systems releasing transmitter such as purines. Such is the case for the ventral horn of the spinal cord. Carlsen and Perrier report on findings in the postnatal mouse that indicate that ventral horn astrocytes produce a tonic and a phasic inhibition of excitatory synaptic transmission in ventral horn neurons by releasing ATP. ATP is rapidly hydrolyzed to adenosine which then acts on presynaptic receptors and thereby decreases the probability of transmitter release. While a role of purinergic processes in synaptic modulation by astrocytes, at least *in vitro*, is established in diverse brain regions, this has been studied in less detail for (ventral) spinal neural circuits.

Maturation of spinal motor circuits are influenced by the development and maintenance of descending serotonergic projections. The review article by Gackière and Vinay describes how 5-HT plays a role in the maturation of locomotor patterning and GABAergic synaptic transmission via actions on 5-HT_2_ and 5-HT_7_ receptors. They describe how postsynaptic inhibition is reduced after SCI and can be accounted for by a 5-HT_2_ receptor-mediated dysregulation of chloride transport in motoneurons. Evidence suggests that 5-HT enables restoration of locomotion after SCI via 5-HT receptors involved in the activation of signal transduction pathways that restore the chloride gradient needed for synaptic inhibition (5-HT_2_) and facilitate interneuronal activity (5-HT_7_).

Sławinska et al. compare the effects of pharmacologic actions of 5-HT_2_ and 5-HT_1A/7_ receptor agonists, applied alone or in combination, on observed recovery of hindlimb treadmill locomotor function after low thoracic spinal transection in adult rats. Assessment of independent drug actions demonstrated that agonists act on complementary circuits: 5-HT_2_ receptors by facilitating motor excitability directly, and 5-HT_1A/7_ receptors by promoting locomotor circuit generating interlimb coordination. These findings add to earlier studies that support combined receptor targeting in locomotor recovery strategies while acknowledging limitations of potentially competing pharmacologic actions on afferent feedback and other intraspinal circuits.

The neurotrophins nerve growth factor (NGF), brain derived neurotrophic factor (BDNF) and neurotrophin-3 (NT-3) play an important role in neural circuit development and plasticity. Neurotrophins and their receptors are normally present in the spinal cord. Boyce and Mendell review how neurotrophic actions modify spinal sensory and motor circuit function including nociception, reflexes and stepping. Experimental interventions have targeted the introduction of exogenous neurotrophins to recapitulate their known trophic actions to improve function in disease states and after injury. This review focuses on more recent findings relevant to these translational issues.

Grau et al. reviews the complex interplay of neuromodulatory factors that alter the capacity for activity-dependent adaptive reflex plasticity (spinal learning) and locomotor recovery after SCI. Adaptive or maladaptive behavioral outcomes are interpreted in relation to prior “priming” events that alter experimental outcome (meta-plasticity). Broadly, adaptive plasticity and motor recovery are tied to an up-regulation of BDNF signaling while maladaptive plasticity and motor deficits are associated with induction of a pro-inflammatory phenotype including expression of the cytokine tumor necrosis factor (TNF). An understanding of spinal cord neuromodulatory status after injury may provide a useful framework for training interventions in the clinical population.

Previous experiments implicate cholinergic brainstem and spinal systems in the control of locomotion. Jordan et al. undertook a pharmacological exploration on the capacity of the spinal cholinergic system in modulating spinal locomotor networks with emphasis on capacity to facilitate recovery after SCI. Contrary to expectations, cholinergic (muscarinic) receptor activation disrupted locomotor recovery while receptor block (atropine) greatly facilitated expression of locomotion and recruitment of cutaneous reflexes. Their temporal correspondence supports the view that there is a tonic muscarinic inhibition of afferent feedback onto locomotor circuits thereby identifying a new opportunity for restoring locomotion after injury.

Spinal motoneurons can exhibit differences in excitability early in development in amyotrophic lateral sclerosis (ALS). As serotonin, noradrenaline, and dopamine already modulate motor activity at early postnatal ages, Milan et al. used the SOD1G93A (SOD1) ALS mouse model to examine possible differences in their expression and function. In ventral horn, there were no differences in HPLC-detected content of these monoamines and metabolites between WT and SOD1 mice (P1 or P10). Similarly, no differences on the locomotor rhythm were observed in the SOD1 mouse when the monoamines were bath applied in the isolated spinal cord (P1–P3). However, NA generated a larger amplitude motor response in SOD1 mice, suggestive of adrenoceptor-based motor excitability increases at early postnatal ages.

## Author Contributions

All authors listed have made a substantial, direct and intellectual contribution to the work, and approved it for publication.

### Conflict of Interest

The authors declare that the research was conducted in the absence of any commercial or financial relationships that could be construed as a potential conflict of interest.

